# More complete polarization of renal tubular epithelial cells by artificial urine

**DOI:** 10.1038/s41420-018-0112-z

**Published:** 2018-10-10

**Authors:** Arada Vinaiphat, Komgrid Charngkaew, Visith Thongboonkerd

**Affiliations:** 1 Medical Proteomics Unit, Office for Research and Development, Faculty of Medicine Siriraj Hospital, Mahidol University, Bangkok, Thailand; 2 Graduate Program in Immunology, Department of Immunology, Faculty of Medicine Siriraj Hospital, Mahidol University, Bangkok, Thailand; 3 Department of Pathology, Faculty of Medicine Siriraj Hospital, Mahidol University, Bangkok, Thailand; 4 Center for Research in Complex Systems Science, Mahidol University, Bangkok, Thailand

**Keywords:** Cell polarity, Proteomics, Proteome, Tight junctions, Kidney

## Abstract

Cell polarization using Transwell is a common method employed to study renal tubular epithelial cells. However, this conventional protocol does not precisely recapitulate renal tubular epithelial cell phenotypes. In this study, we simulated renal physiological microenvironment by replacing serum-containing culture medium in upper chamber of the Transwell with physiologic artificial urine (AU) (to mimic renal tubular fluid), whereas the lower chamber still contained serum-containing medium (to mimic plasma-enriched renal interstitium). Comparing to the conventional protocol (control), the AU-assisted protocol offered more complete polarization of MDCK renal tubular cells as indicated by higher transepithelial electrical resistance (TER) and greater levels of tight junction (TJ) proteins (ZO-1 and occludin). Transmission electron microscopy (TEM) showed greater densities of TJ and desmosome, narrower intercellular spaces, greater cell height, and longer microvilli in the AU-treated cells. Secretome analysis revealed that the AU-treated cells secreted greater proportion of the proteins matched to normal human urinary proteome via both classical and non-classical secretory pathways. Finally, modifying/omitting each component of AU (one at a time) followed by validation revealed that urea was responsible for such property of AU to improve cell polarization. These data indicate that replacing AU on the upper chamber of Transwell can improve or optimize renal cell polarization for more precise investigations of renal physiology and cell biology in vitro.

## Introduction

Mammalian cell culture has served as a fundamental tool for in vitro studies of cell and molecular biology for several decades. It offers several advantages to complement in vivo investigations, mainly because of its ease to access, control, and manipulate. For these reasons, researchers can measure alterations in cells upon treatment with high reproducibility and accuracy^1^. To study renal physiology and pathophysiology, cell lines are frequently grown on culture plates, Transwell, chemotaxis chamber, etc. Numerous studies have shown that these convenient and simple culture systems can be applied to study sophisticated mechanisms of kidney diseases^2–5^. In the case of Transwell culture system, its specialized feature for polarized cell cultivation that can separate upper and lower chambers allows independent access to both distinct fluid compartments and offers advantages for evaluation of cellular functions relating to apico-basolateral trans/paracellular transports^6,7^. Moreover, the Transwell culture system is also beneficial for co-culture studies^8^. Although the polarized cell culture system is an invaluable tool for basic cell biology research, only around 10% of the data derived from this in vitro system successfully go through clinical applications and drug development due to its inadequate precision to recapitulate the in vivo microenvironment of the cells^9,10^. Therefore, it is imperative to develop a better polarized cell culture system that more closely emulate the physiology of renal tubular epithelial cells.

One among the main functions of the kidney is to remove metabolic waste products from blood circulation, while controlling appropriate water-solutes balance to maintain body homeostasis by concentrating the urine^11^. By both anatomical and physiological aspects, renal tubular epithelial cells are exposed to different body fluids. Their basolateral compartment is exposed to the plasma (via renal interstitial microcirculation), whereas apical segment (which faces to tubular lumen) is directly exposed to renal tubular fluid or the urine. While conventional polarized cell culture system normally uses serum-containing culture medium in both upper and lower chambers of Transwell, we hypothesized that replacing the serum-containing culture medium in upper chamber with artificial urine (AU) would be more physiologic to the cells. Our hypothesis was then confirmed by several cell biology and functional assays.

## Results

### Effects of AU on transepithelial electrical resistance (TER)

**Fig. 1:**
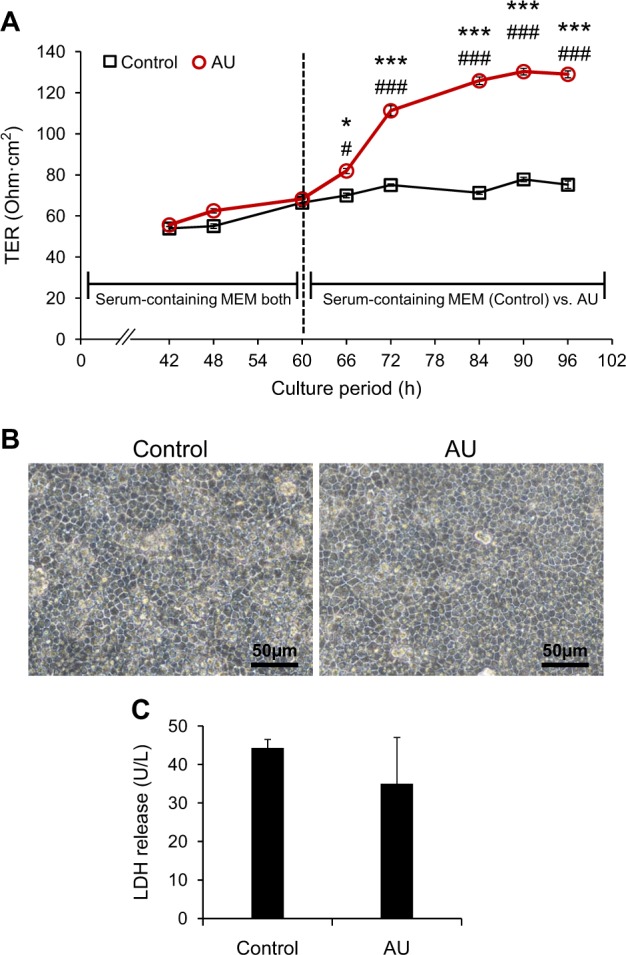
Effects of AU on transepithelial electrical resistance (TER). **a** MDCK cells were first grown in 12-mm Transwell with serum-containing MEM in both upper and lower chambers. From 42-h post-culture onward, TER was periodically measured. At 60-h post-culture, when TER was no longer increased and could be stabilized, serum-containing medium in the upper chamber was then replaced with physiologic AU, whereas the cells that were still maintained in serum-containing medium (conventional culture) served as the controls. At 24-h after switching culture medium in the upper chamber (or 84-h post-culture), when TER of the AU group reached its plateau and was no longer increased, the cells were then subjected to evaluations by various assays, e.g., morphological examination under a light microscope (**b**) and measurement of LDH released into the culture medium (**c**). Each bar or dot represents mean ± SEM of the data obtained from three independent biological replicates. *p < 0.05 vs. control at each corresponding time-point; ***p < 0.001 vs. control at each corresponding time-point; #*p* < 0.05 vs. AU at 60-h; ###*p* < 0.001 vs. AU at 60-h

TER was assessed as an indicator for status of epithelial cell differentiation and barrier function of tight junction (TJ) and other paracellular junctions^12^. The data showed that TER, which was started to be measured at 42-h post-culture (when the polarized monolayer started to form), was gradually increased along the incubation time-points. At 60 h, when TER was no longer increased and could be stabilized (Fig. 1a), the serum-containing culture medium in the upper chamber was either refreshed (control) or switched to AU. After switching the culture medium in the upper chamber, TER of the AU-treated cells was dramatically increased and reached its plateau at 84-h post-culture (or 24-h after switching the culture medium) (approximately 1.8-fold increase as compared to its basal level at 60-h post-culture or before switching the culture medium), whereas that of the controlled cells remained unchanged (Fig. 1a). This time-point (84-h post-culture or 24-h post-switching) was then used for all subsequent assays. After such switching, there were no significant differences in cell morphology and level of lactate dehydrogenase (LDH) released from the two groups of cells (Fig. 1b, c).

### Effect of AU on expression of TJ and adherens junction (AJ) proteins

**Fig. 2:**
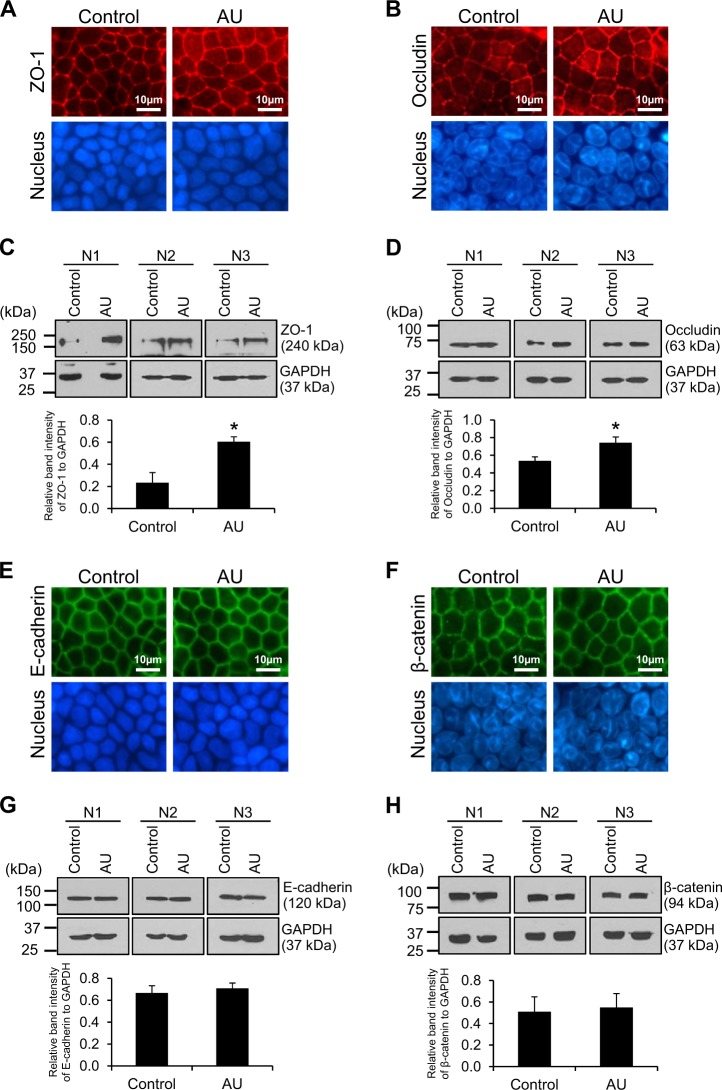
Effect of AU on expression of TJ and AJ proteins. After switching culture medium in the upper chamber for 24 h, the controlled and AU-treated cells were subjected to immunofluorescence staining and Western blot analysis of markers for TJ (ZO-1 and occludin) (**a**-**d**) and markers for AJ (E-cadherin and β-catenin) (**e**-**h**). Note that GAPDH served as the loading control and full-size scans of membranes of these cropped images are shown as Supplementary Figure S1. Each bar represents mean ± SEM of the data obtained from three independent biological replicates. *p < 0.05 vs. control

After switching culture medium in the upper chamber for 24 h, the controlled and AU-treated cells were subjected to immunofluorescence staining and Western blotting for markers of TJ (zonula occludens-1 or ZO-1 and occludin) and AJ (E-cadherin and β-catenin) complexes. The data showed that expression levels of both TJ markers were significantly increased in the AU-treated cells (Fig. 2a–d), whereas those of AJ markers remained unchanged (Fig. 2e–h) (see also Supplementary Figure S1 for the full-size scans of membranes).

### Effect of AU on TJ density, desmosome density, and intercellular space

**Fig. 3:**
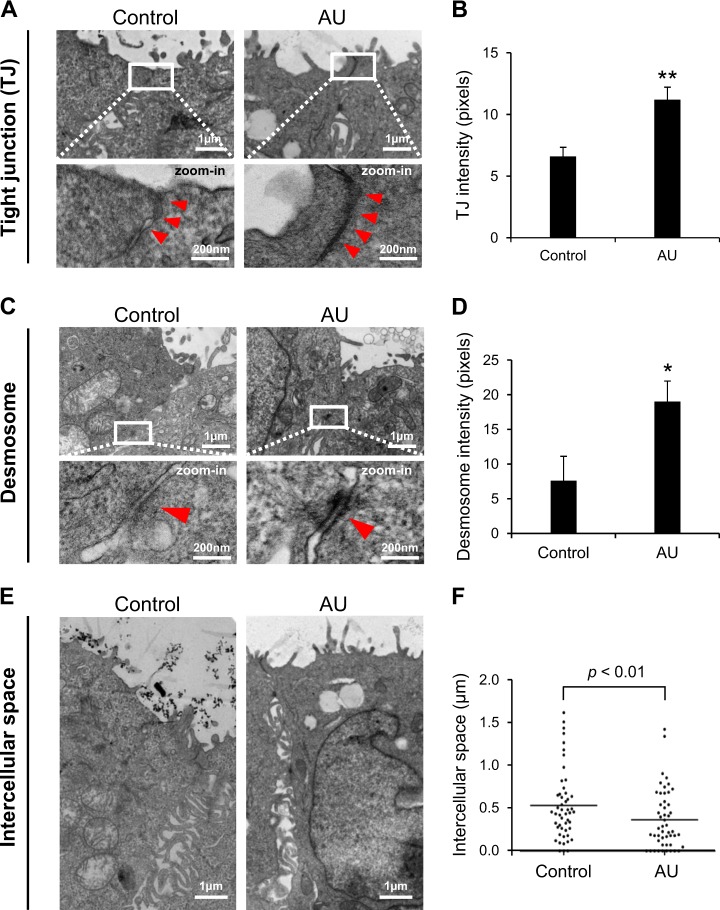
Effect of AU on TJ density, desmosome density, and intercellular space. After switching culture medium in the upper chamber for 24 h, the controlled and AU-treated cells were subjected to examination by transmission electron microscopy (TEM). Quantitative analysis was performed on the ultra-thin sections to measure densities of TJ (**a** and **b**) and desmosome (**c** and **d**) (both are marked with arrow heads), and intercellular space (**e** and **f**). Each bar represents mean ± SEM of the data obtained from three independent biological replicates, whereas dot plot represents mean ± SEM of the data obtained from at least 50 measurements in ≥ 50 cells from ≥ 5 different ultra-thin sections in each group. *p < 0.05 vs. control

After switching culture medium in the upper chamber for 24 h, the controlled and AU-treated cells were subjected to examination by transmission electron microscopy (TEM). Whereas the cellular ultrastructure looked comparable, quantitative analysis of the TEM data revealed that the AU-treated cells had more intense TJ and desmosome as compared to the controls (Fig. 3a–d). Additionally, the AU-treated cells had narrower intercellular space or gap as compared to the controls (Fig. 3e, f).

### Effect of AU on epithelial cell microstructure and ultrastructure

**Fig. 4:**
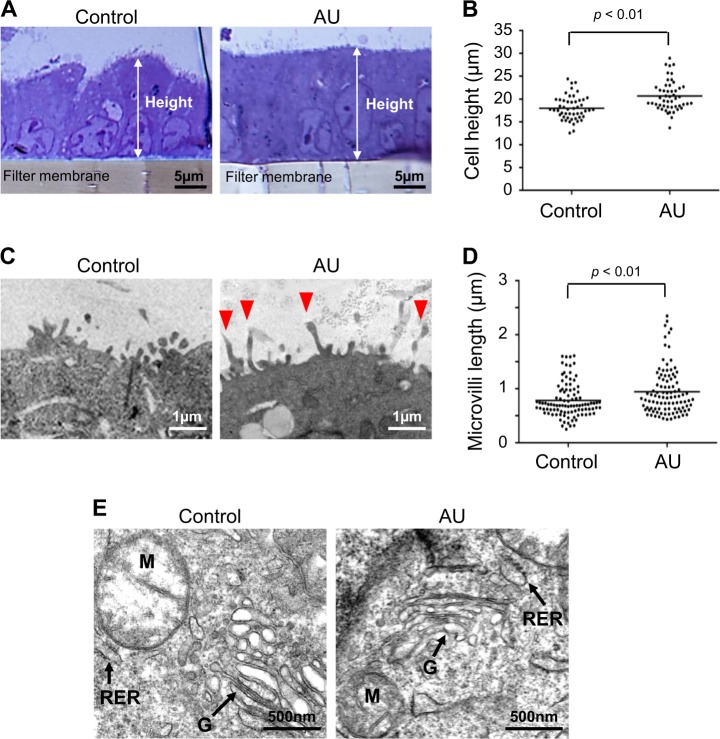
Effect of AU on epithelial cell microstructure and ultrastructure. After switching culture medium in the upper chamber for 24 h, the controlled and AU-treated cells were subjected to examination by transmission electron microscopy (TEM). Quantitative analysis was performed on the thick sections to measure cell height (**a**, **b**) and length of the microvilli (marked with arrow heads) (**c**, **d**). Evaluation of the intracellular organelles was performed on the ultra-thin sections (**e**). Each dot plot represents mean ± SEM of the data obtained from at least 50 measurements in ≥50 cells from ≥5 different thick sections in each group. M mitochondria, RER rough endoplasmic reticulum, G Golgi apparatus

In addition to TJ, desmosome, and intercellular space, microstructure and ultrastructure of the cells were also evaluated. The data showed that cell height and microvillar length were significantly increased by AU treatment (Fig. 4a–d), whereas intracellular organelles appeared unchanged (Fig. 4e).

### Secretome analysis

After switching culture medium in the upper chamber for 24 h, the cells were washed with PBS^+^ three times and the serum-containing culture medium in both upper and lower chambers of the controlled group and in the lower chamber of the AU group was replaced with the serum-free medium (to eliminate contaminants from serum proteins), whereas AU in the upper chamber of the AU group was refreshed. The cells were further incubated for additional 12 h (at 96-h post-culture), at which TER and its differences between groups remained unchanged as compared to the former time-point (at 84-h post-culture) (Supplementary Figure S2). The culture supernatants from upper chambers were then collected and subjected to secretome analysis. Only the proteins that were identified from at least two out of three biological replicates in each group are reported herein (Supplementary Table S1). After subtracting proteins that were identified in both groups, differentially secreted proteins that were exclusively present in each group were further analyzed.

**Fig. 5:**
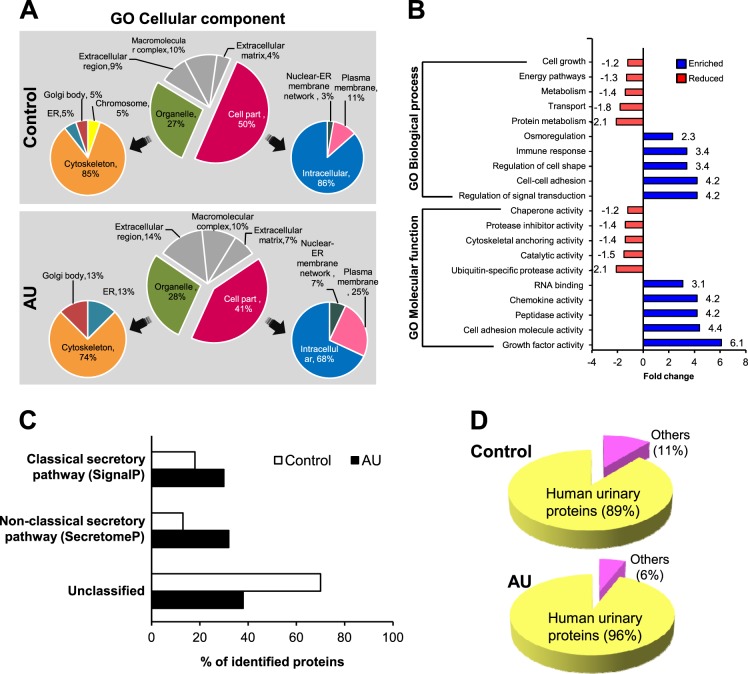
Secretome analysis. Schematic diagram of the study design and time-point selected for sample collection are illustrated as Supplementary Figure S2. The culture supernatants from upper chambers were then collected and subjected to secretome analysis (see also Supplementary Table S1). After subtracting proteins that were identified in both groups, differentially secreted proteins that were exclusively present in each group were further analyzed for GO cellular component (**a**), biological process and molecular function (**b**), and secretory pathways (**c**). Moreover, these differentially secreted proteins were also matched with normal human urinary proteins identified from 23 previous large-scale proteomics studies^13–35^ (**d**)

Based on gene ontology (GO) cellular component, these differentially secreted proteins were classified into six categories, of which the two largest groups, ‘organelle’ and ‘cell part’, were further highlighted (Fig. 5a). The data showed that proteins originated from ‘plasma membrane’ and ‘extracellular region’ were enriched in the AU group, whereas those originated from ‘cytoskeleton’ and ‘intracellular’ compartments were predominant in the controlled group (Fig. 5a). In addition, GO biological process and molecular function were analyzed, and top-ten enriched and depleted processes/functions based on GO of the AU dataset comparing to the control were generated (Fig. 5b). The data showed that three groups of proteins directly associated with establishment and maintenance of cell polarity, including regulation of cell shape, cell-cell adhesion, and cell adhesion molecule activity, were enriched in the AU group (Fig. 5b). Analysis of secretory pathways of these differentially secreted proteins revealed that the AU-treated cells secreted greater percentages of proteins via both classical and non-classical pathways, whereas the control secreted proteins mainly via the unclassified pathway (Fig. 5c). To evaluate whether renal tubular cells under in vitro AU-assisted polarized cell culture system could closely mimic the in vivo renal cell features, the identified secreted proteins were matched with human urinary proteome identified from 23 previous large-scale proteomics studies^13–35^. Figure 5d demonstrates that the AU-treated cells secreted greater proportion of the proteins matched with human urinary proteins, implicating that the cells derived from AU-assisted culture condition might better mimic renal tubular cells in vivo.

### Effect of AU on cellular uptake or secretion of various ions and solutes

**Fig. 6:**
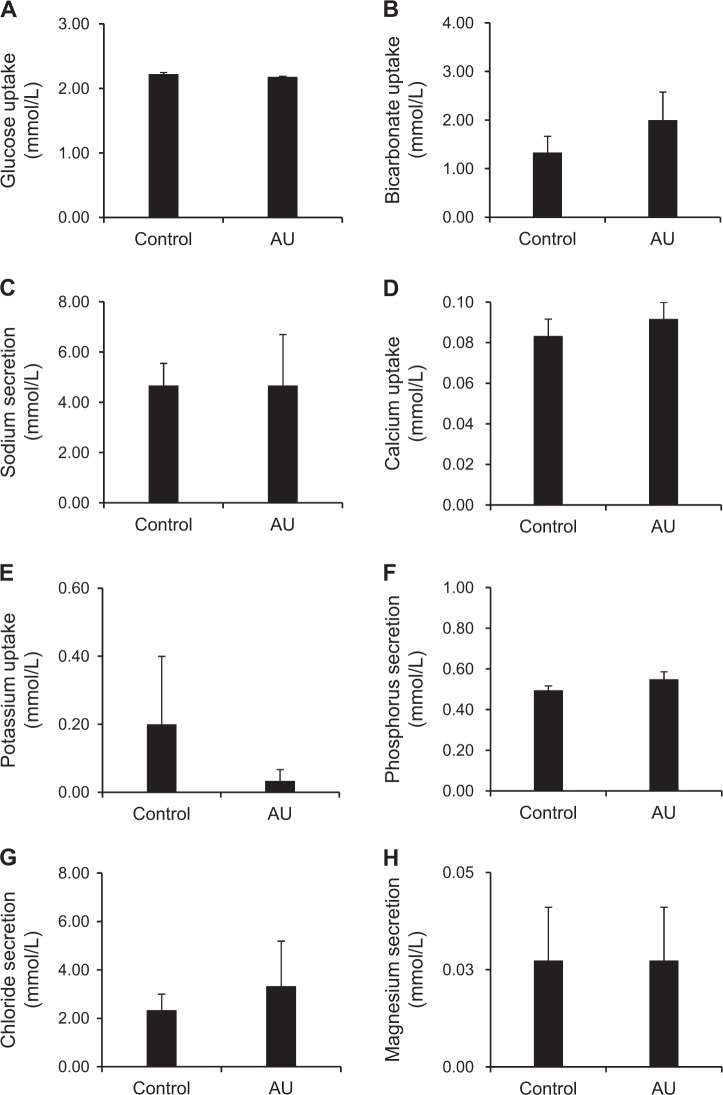
Effect of AU on cellular uptake or secretion of various ions and solutes. After switching culture medium in the upper chamber for 24 h (at 84-h post-culture), the controlled and AU-treated cells were washed three times with PBS^+^ and serum-containing culture medium in the lower chamber was refreshed, whereas that in the upper chamber was replaced with PBS^+^. The cells were further incubated at 37 °C for 6 h and the resulting supernatants from both upper and lower chambers of each well were subjected to measurements for levels of various ions and solutes, i.e., glucose (**a**), bicarbonate (**b**), sodium (**c**), calcium (**d**), potassium (**e**), phosphorus (**f**), chloride (**g**), and magnesium (**h**). Their levels at 6-h post-incubation (90-h post-culture) were then subtracted with their basal levels (at 84-h post-culture) measured using the same technique. The positive values denoted cellular secretion, whereas the negative values indicated the cellular uptake. Each bar represents mean ± SEM of the data obtained from three independent biological replicates

The separation of apical and basolateral compartments of the polarized cells allows specialized cellular vectorial transports (either uptake or secretion) of various ions and solutes. Such vectorial transports of glucose, bicarbonate, sodium, calcium, potassium, phosphorus, chloride and magnesium were then evaluated. The data showed that there were no significant differences in vectorial transports of these ions and solutes observed in the controlled and AU-treated cells (Fig. 6).

### Identification of essential composition of AU that was responsible for the improvement of cell polarization

**Fig. 7:**
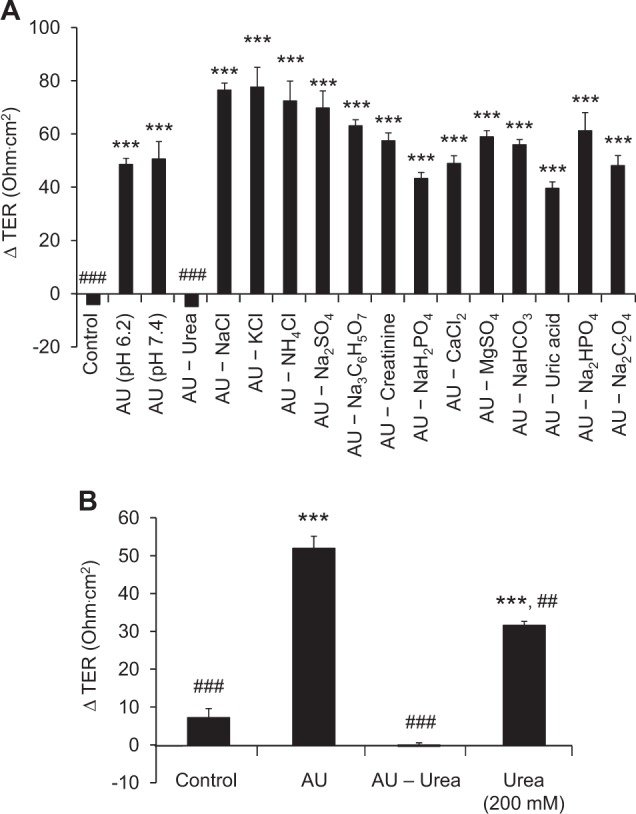
Identification of essential composition of AU that was responsible for the improvement of cell polarization. Each composition of the AU was evaluated, one at a time, for its effect on ΔTER as calculated from subtraction of the TER at 84-h post-culture (after switching culture medium in the upper chamber for 24 h) from that measured at 60-h post-culture (before switching culture medium in the upper chamber) (**a**). ‘AU – X’ represents the absence of X in the AU formula. To confirm the essential role of urea, the ΔTER was again evaluated by using 200 mM urea when switching the culture medium in the upper chamber, comparing to serum-containing medium (control), standardized AU (pH 6.2; containing 200 mM urea), and ‘AU – Urea’ (AU without any urea) (**b**). Each bar represents mean ± SEM of the data obtained from three independent biological replicates. *** *p* < 0.001 vs. control; ## *p* < 0.01 vs. AU (pH 6.2); ### *p* < 0.001 vs. AU (pH 6.2)

Each composition of the AU was evaluated for its effect on ΔTER one at a time. The one that was modified or omitted from the specialized AU and caused obvious decrease in ΔTER as calculated from subtraction of the TER at 84-h post-culture (after switching culture medium in the upper chamber for 24 h) from that measured at 60-h (before switching culture medium in the upper chamber) would be considered as the essential composition responsible for the improvement of cell polarization. The data showed that omitting urea from the AU caused obvious decrease in ΔTER to the same level as of the control, whereas modifying or omitting other compositions had no such obvious change (Fig. 7a), indicating that urea in AU might be responsible for such improvement.

To address such hypothesis, the ΔTER was again evaluated by using 200 mM urea to replace the culture medium in the upper chamber, comparing to serum-containing medium (control), standardized AU (pH 6.2; containing 200 mM urea), and ‘AU – Urea’ (AU pH 6.2 without any urea). The data showed that while ‘AU – Urea’ caused significant decrease in ΔTER as compared to the standardized AU, 200 mM urea induced much greater ΔTER as compared to the ‘AU – Urea’ (although still less than that of the standardized AU) (Fig. 7b). This might implicate that urea was responsible for the improvement of cell polarization. However, urea alone could not resemble all features of the AU that is required for the more complete cell polarization.

## Discussion

The aim of this study was to develop a more physiologically relevant polarized cell culture system for renal tubular epithelial cells using MDCK cell line. Initial assessment of the effects of AU on cells grown in Transwell demonstrated that the AU-treated cells exhibited greater barrier function of the polarized cells as indicated by greater TER, whereas the cells looked healthy without any signs of cytotoxicity (Fig. 1). The lack of the cytotoxic effects was most likely due to the availability of recently established AU protocol that resembles more closely to the normal or physiologic urine in healthy individuals^36,37^. In consistent with the greater TER, which is known to be affected mainly by TJ integrity^12,38^, the AU-treated cells showed significantly increased levels of TJ proteins (ZO-1 and occludin) as determined by immunofluorescence staining and Western blotting (Fig. 2a–d) and greater density of TJ as determined by TEM (Fig. 3a, b). In addition, the AU-treated cells showed more intense desmosome as demonstrated by TEM as a dense and well-defined network of tonofibrils or cytokeratins^39^ extended from the adhesion plaque (desmoplakin) into the cell cytoplasm on either side of the paracellular junction (Fig. 3c, d). Moreover, these features of the AU-treated cells were accompanied by the narrower intercellular space as measured from the TEM views (Fig. 3e, f). The tightening of TJ and intercellular junction along with the increases in cellular height and microvillar length (Fig. 4a–d) indicated that the cells underwent more complete polarization in the AU-assisted protocol.

Cellular morphology is one among several characteristics indicating degree of cell polarization. Several studies have shown decreases in number and length of microvilli to indicate the defect or loss renal epithelial cell polarity induced by ischemic, oxidative, or other stresses^4,40–42^. Similarly, studies on different types of epithelial cells have shown that the defect or loss of cell integrity is correlated with the decrease of cell height^43,44^ and the increase of cellular junction distance (intercellular space)^45^. Nevertheless, the correlation between increased/enhanced degree of cell polarity and protrusion/elongation of microvilli, cell height, or intercellular space had been previously under-investigated. Our present study highlighted such significance of cell morphology and utilized it as a tool to evaluate the improvement of cell polarization by AU.

Several recent studies have shown that cellular proteome and secretome compositions as well as their changes can reflect biological processes and physiological status of the cells^34,46^. In this study, we also performed secretome analysis to characterize secreted proteins from the AU-treated vs. controlled cells aiming for defining potential effect of different degree of polarization on secretion of proteins from renal tubular epithelial cells. The hypothesis was that proteins secreted into the apical chamber of Transwell from the more complete polarized renal tubular epithelial cells should better mimic the normal urinary proteins. Our data confirmed that the AU-treated cells secreted larger proportion of normal human urinary proteins into the apical chamber as compared to the control (Fig. 5d). And the sources of such secretory proteins were mainly from ‘plasma membrane’ and ‘extracellular region’ (Fig. 5a).

Secretory pathway prediction by SignalP and SecretomeP tools demonstrated the enrichment of proteins containing signal peptides for classical pathway secretion and leaderless proteins that secreted through non-classical pathway in the AU-treated cells (Fig. 5c). In contrast, proteins that fell within the ‘unclassified’ group were found as the most abundant proteins secreted from the controlled polarized cells (Fig. 5c). The ‘unclassified’ secretome could possibly be a group of proteins in which their secretory pathways have not yet been discovered or a group of intracellular proteins released from a nonlethal, mechanically wounded cells, e.g., those induced by washing and/or cell manipulation steps^47–49^. On the other hand, the smaller proportion of the unclassified secretory proteins found in the AU group might suggest that the AU-assisted polarized epithelial cells, to some extent, had greater integrity as the cells were better preserved as reflected by the less degree of contamination by these unclassified secretory proteins, strengthening the role of AU to improve the integrity of polarized cell monolayer to better tolerate to mechanical force imposed during secretome collection.

In concordance to the notion that the AU-treated cells exhibited better mechanical stress tolerance, differentially secreted proteins classified by GO analysis with respect to subcellular localization demonstrated that extracellular locales of the proteins, i.e., ‘extracellular region’, ‘extracellular matrix’, and ‘plasma membrane’, were found with greater proportion in the secretome derived from the AU-treated cells (Fig. 5a). Moreover, the result obtained from enrichment analysis by GO biological process and molecular function revealed that proteins mediating cell shape and cell-cell adhesion were enriched by 3–4 folds in the AU group comparing to the control (Fig. 5b). The increase in these proteins involved in regulation of epithelial cell architecture also confirmed that TJ and intercellular junctional complexes of the polarized cell monolayer could be strengthened by our AU-assisted protocol.

Functional apico-basolateral polarity of epithelial cells was also examined. The distribution and targeting of transportors/channels to the distinct plasma membrane domains in polarized epithelial cells is critical for vectorial transports of ions and solutes. While the TJ and intercellular junctional complexes of the polarized cell monolayer could be strengthened by AU, we also demonstrated that the AU-treated cell monolayer could still perform cellular vectorial transports of various ions and solutes comparable to the control without any problems (Fig. 6).

Finally, mechanism by which AU modulated cell polarization and integrity was investigated by modifying/omitting each of the ions and solutes one at a time. Urea (averaging 200 mM in the normal physiologic urine) accounted for most of the AU solute constituents. The absence of urea in the AU led to a profound decrease of osmolality of the AU (from 446 mOsm/kg to 246 mOsm/kg). As a result, the exclusion of urea from the AU markedly diminished ΔTER to the level that was comparable to the control, whereas using 200 mM urea alone (in replacement of the AU) could increase ΔTER although not to the same level as of the AU (Fig. 7).

The epithelial lining of the nephron comprises specialized single cell monolayer with ability to adapt and maintain its proper function under high osmotic pressure inside tubular lumen caused by excretion of various ions and solutes, in particular urea^50^. The role of urea in the urine concentrating mechanism has been well-documented^11^. Earlier studies have reported that renal epithelial cells, MDCK and LLC-PK1, responded to hyperosmotic state induced by 200 mM urea by triggering expression of specific set of genes that differed from those induced by sodium and mannitol, both of which are the other potent osmotic agents^50,51^. Importantly, urea at <400 mM neither caused adverse effects on cellular function nor induced transcription of stress-related genes as observed in NaCl-induced hyperosmotic stress^52–54^. In concordance to our results, a previous study has reported interesting findings showing that the inner medulla collecting duct (IMCD) cells had increased levels of proteins associated with TJ integrity upon exposure to sublethal hypertonic state (550 mOsm/kg)^55^. Taken together, these data support our hypothesis that MDCK cells had an adaptive response to tolerate with hyperosmotic state (osmotolerance) caused by various ions and solutes in the AU. However, only urea caused significant upregulation of the proteins involved in integrities of TJ and other intercellular junctional complexes^55^, and thereby strengthened these junctional protein complexes and improved the cell polarization.

In summary, this study has shown that using AU in the upper chamber of the Transwell could improve polarization of renal tubular epithelial cells by simulating or mimicking the microenvironment that usually exposes to renal tubular cells in vivo. Such improvement should make the *in vitro* investigations of cell biology and physiology of the renal tubular epithelial cells more precise and more reliable.

## Materials and methods

### Preparation of physiologic AU

Fresh physiologic AU solution was prepared as described previously^36,37^. The final compositions of this AU included 200 mM urea, 1 mM uric acid, 4 mM creatinine, 5 mM Na_3_C_6_H_5_O_7_·2H_2_O, 54 mM NaCl, 30 mM KCl, 15 mM NH_4_Cl, 3 mM CaCl_2_·2H_2_O, 2 mM MgSO_4_·7H_2_O, 2 mM NaHCO_3_, 0.1 mM NaC_2_O_4_, 9 mM Na_2_SO_4_, 3.6 mM NaH_2_PO_4_·H_2_O, and 0.4 mM Na_2_HPO_4_. Final pH, specific gravity, and osmolality of this physiologic AU were 6.2, 1.010 (g/ml), and 446 (mOsm/kg), respectively.

### Polarized cell culture: conventional (control) protocol vs. AU-assisted protocol

Madin-Darby Canine Kidney (MDCK) cell line (strain II) (ATCC; Manassas, VA), the most commonly used strain for in vitro epithelial cell polarity studies^56,57^, was employed. The cells at a density of 7.5 × 10^4^ cells per 1.12-cm^2^ area were seeded on a collagen-coated polyethylene culture insert of the 12-mm Transwell (0.4-µm pore size) (Corning Costar; Cambridge, MA) and grown under standard condition in Eagle’s minimum essential medium (MEM) (Gibco, Invitrogen; Grand Island, NY) supplemented with 10% heat-inactivated fetal bovine serum (FBS) (Gibco), 2 mM L-glutamine (Sigma; St. Loius, MO), 60 U/ml penicillin G (Sigma), and 60 mg/ml streptomycin (Sigma) in a humidified incubator containing 5% CO_2_ at 37 °C. The medium was refreshed every other day.

The cells were maintained in serum-containing MEM for 60 h, when transepithelial electrical resistance (TER) was no longer increased and could be stabilized. The serum-containing medium in upper chamber was then replaced with physiologic AU, whereas the cells that were still maintained in serum-containing medium (conventional culture) served as the controls. After switching culture medium in the upper chamber for 24 h (or at 84-h post-culture), the cells were subjected to several various investigations as follows.

### TER measurement

Tight junction (TJ) integrity was investigated as one of the indicators for the completion of the cell polarization. The polarized MDCK cells were subjected to TER measurement at three different sites in each sample well using Millicell-ERS resistance system (Millipore; Bedford, MA)^58,59^. The resistance value obtained from the sample well was then subtracted with the background value obtained from the blank control (coated-well without cells filled with the same conditioned medium).

### Immunofluorescence staining

After switching culture medium in the upper chamber for 24 h, the cells were subjected to immunofluorescence staining for markers of tight junction (TJ) and adherens junction (AJ)^60,61^. Briefly, the cells were washed three times with membrane preserving buffer (or PBS^+^) (1 mM MgCl_2_ and 0.1 mM CaCl_2_ in PBS), fixed with 4% paraformaldehyde in PBS at 25 °C for 15 min, and permeabilized with 0.1% Triton X-100 in PBS at 25 °C for 15 min. Non-specific bindings were blocked with 1% BSA in PBS at 25 °C for 30 min. After washing with PBS^+^, the cells were incubated with mouse monoclonal anti-ZO-1 (Invitrogen-Molecular Probes; Eugene, OR), rabbit polyclonal anti-occludin (Santa Cruz Biotechnology; Santa Cruz, CA), rabbit polyclonal anti-E-cadherin (Santa Cruz Biotechnology), or mouse monoclonal anti-β-catenin (Santa Cruz Biotechnology) primary antibody (all were diluted 1:50 in 1% BSA/PBS) at 37 °C for 1 h. After another wash, the cells were incubated with Alexa546- conjugated or Alexa488-conjugated secondary antibody (Invitrogen-Molecular probes) (1:500 in 1% BSA/PBS) containing 0.1 g/ml Hoechst dye (Sigma) at 37 °C for 1 h. Finally, the cells were washed with PBS^+^ and mounted onto glass slides using 50% glycerol/PBS for subsequent examination by using a fluorescence microscope (Eclipse Ti-S, Nikon; Tokyo, Japan).

### Western blotting

After switching culture medium in the upper chamber for 24 h, proteins were extracted from the controlled or AU-treated cells using Laemmli’s buffer and quantified by Bradford’s method using Bio-Rad Protein Assay (Bio-Rad Laboratories; Hercules, CA). Thereafter, equal amount of total protein was resolved by 12% SDS-PAGE (30 µg/lane) and the resolved proteins were transferred onto a nitrocellulose membrane. Non-specific bindings were blocked with 5% skim milk/PBS and the membrane was then incubated with mouse monoclonal anti-ZO-1 (Invitrogen-Molecular Probes), rabbit polyclonal anti-occludin (Santa Cruz Biotechnology), rabbit polyclonal anti-E-cadherin (Santa Cruz Biotechnology), mouse monoclonal anti-β-catenin (Santa Cruz Biotechnology), or mouse monoclonal anti-GAPDH (Santa Cruz Biotechnology) primary antibody (all were diluted 1:1000 in 1% skim milk/PBS) at 4 °C overnight. After washing with PBS, the membrane was further incubated with corresponding secondary antibody conjugated with horseradish peroxidase (1:2000 in 1% skim milk/PBS) at 25 °C for 1 h. Immunoreactive protein bands were visualized by SuperSignal West Pico chemiluminescence substrate (Pierce Biotechnology, Inc.; Rockford, IL) and autoradiography. Band intensity data was obtained using ImageQuant TL software (GE Healthcare; Uppsala, Sweden).

### Transmission electron microscopy (TEM)

After switching culture medium in the upper chamber for 24 h, the controlled and AU-treated cells were subjected to examination by TEM. The cells were washed three times with ice-cold Millonig’s phosphate buffer (NaH_2_PO_4_·H_2_O, Na_2_HPO_4_·H_2_O, and 0.5% NaCl; pH 7.4) and then fixed with 2.5% glutaraldehyde for 1 h followed by 1% osmium tetroxide (OsO_4_) for 30 min. The cells were then incubated with 2% uranyl acetate in the dark for 20 min and then dehydrated through graded ethanol series of 30, 50, 70, 90 and 100%. Epoxy and ethanol solution (1:1 v/v) was then added to both chambers and incubated for 1 h to allow infiltration, followed by 2-h pre-polymerization of the cell monolayer in full epoxy resin (100%) at 37 °C. Epoxy resin was then discarded and replaced with fresh resin, which was polymerized at 65 °C for 16 h in a hot air oven. The resin-embedded cell monolayer was then excised into a 4 × 4 × 4 mm cubical block based on protocol described previously^62^. The block was then set up on the microtome to obtain thick (1-µm) and ultra-thin (50-nm) sections. The thick sections were heat-fixed on glass slides and stained with toluidine blue solution for 2–3 min followed by three washes with deionized water. These thick sections were then examined by using a phase-contrast mode of Nikon Eclipse Ti-S microscope with magnification power of 1000 × . The ultra-thin sections were fixed on copper grids and were contrasted with 2% uranyl acetate for 15 min. After a gentle wash with deionized water and air-dried, the cells on grids were stained with Reynolds’ lead citrate for 5 min followed by another wash. The processed ultra-thin sections were then examined by using a transmission electron microscope (FEI Technai G2 twin; Hillsboro, OR) operated at 200 kV with magnification power of 40,000×.

### Measurements of cell height, microvilli length, TJ density, desmosome density, and intercellular space

At least 50 measurements were performed in ≥ 50 cells from ≥ 5 different sections for each group. Cell heights were measured from thick sections using NIS-Element D software v 4.11 (Nikon). TEM images derived from ultra-thin sections were then subjected to other measurements. Microvilli lengths were measured using NIS-Element D software v 4.11, whereas TJ density, desmosome density, and intercellular space were measured using ImageMaster 2D Platinum software (GE Healthcare; Uppsala, Sweden) comparing to an internal scale marker. Note that each measurement of the intercellular space was averaged from ten equally distributed transect lines along the intercellular space of each pair of the two adjacent cells.

### Measurements of cellular uptake or secretion of various ions and solutes, and lactate dehydrogenase (LDH) release

After switching culture medium in the upper chamber for 24 h, the controlled and AU-treated cells were washed three times with membrane preserving buffer (PBS^+^) (1 mM MgCl_2_ and 0.1 mM CaCl_2_ in PBS) and the serum-containing MEM culture medium in the lower chamber was refreshed, whereas that in the upper chamber was replaced with PBS^+^ to generate the ion gradient for ion transport evaluation. The cells were further incubated at 37 °C for 6 h and the resulting supernatants from both upper and lower chambers of each well were subjected to measurements for levels of various ions and solutes, i.e., glucose, bicarbonate, sodium, calcium, potassium, phosphate, chloride, and magnesium, as well as LDH using Architect c16000 Clinical Chemistry Autoanalyzer (Abbott Diagnostics Ltd; Abbott Park, IL). Their levels at 6-h post-incubation (90-h post-culture) were then subtracted with their basal levels (at 84-h post-culture) measured using the same technique. The positive values denoted cellular secretion, whereas the negative values indicated cellular uptake.

### Identification of the essential composition of AU responsible for the improvement of cell polarization

To define the critical composition of the AU that was responsible for the improved polarization of the cells, each of the AU composition was adjusted or omitted one at a time. First, pH of the AU was adjusted from 6.2 to 7.4 using NaOH. In addition, the specialized AU (‘AU – X’) was generated by omitting each of the AU compositions one at a time. For example, ‘AU – Urea’ meant this specialized AU was generated in absence of urea, whereas other compositions remained the same as of the standardized physiologic AU (pH 6.2). TER of the cell monolayer was measured at 60-h post-culture, just before switching the culture medium in the upper chamber, and at 84-h post-culture or 24-h post-switching using either serum-containing medium (control), standardized AU (pH 6.2), AU (pH 7.4), and various specialized ‘AU – X’ protocols. ΔTER obtained from each of these AU protocols was then calculated using the following formula:

Formula 1: ΔTER (Ohm·cm^2^) = TER after 24-h switching (at 84-h post-culture) – TER before switching (at 60-h post-culture)

### Sample preparation for secretome analysis

Approximately 6.5 × 10^5^ MDCK cells were seeded in each of a large-size (100-mm) Transwell (0.4-µm pore size) (Corning Costar). After switching culture medium in the upper chamber for 24 h, the cells were washed with PBS^+^ three times and the serum-containing medium in upper and lower chambers of the controlled group and in the lower chamber of the AU-treated group was replaced with serum-free MEM (to eliminate contaminants from serum proteins), whereas the upper chamber of the AU-treated group was refreshed with AU. The cells were further incubated at 37 °C for 12 h. Finally, the resulting supernatants from the upper chambers of both groups were collected, transferred to 15-ml conical tubes, and centrifuged at 10,000 × *g* and 4 °C for 10 min. The clear supernatants were then subjected to secretome analysis^63,64^.

### In-solution tryptic digestion by filter-aided sample preparation (FASP) method

Protein samples prepared in SDT lysis buffer (4% SDS, 100 mM DTT, and 100 mM Tris-HCl; pH 7.6) were reduced by heating at 95 °C for 5 min. After cooling down at 25 °C, 30–100 µg of each protein sample was transferred to an Omega Nanosep 10 K device (Pall Corporation; Port Washington, NY), added with 200 µl of 8 M urea in 100 mM Tris-HCl (pH 8.5), and then centrifuged at 14,000 × *g* and 25 °C for 15 min. This buffer exchange step was repeated one more cycle. The recovered proteins were then alkylated with 100 µl of 50 mM iodoacetamide in 8 M urea/100 mM Tris-HCl (pH 8.5) at 25 °C in the dark using a ThermoMixer^®^ C (Eppendorf; Hauppauge, NY) for 20 min. Thereafter, buffer exchange was performed twice by centrifugation at 14,000 × *g* and 25 °C for 15 min each using 200 µl of 8 M urea/100 mM Tris-HCl (pH 8.5). The proteins were then finally exchanged into 50 mM NH_4_HCO_3_ and digested with sequencing grade modified trypsin (Promega; Madison, WI) in 50 mM NH_4_HCO_3_ at a ratio of 1:50 (w/w) trypsin/protein at 37 °C for 16–18 h in a ThermoMixer^®^ C. The digested peptides were collected by transferring the filter unit to a new collection tube and centrifuged at 14,000 × *g* and 25 °C for 10–20 min. Trypsin activity was then stopped by adding 10 µl of 5% formic acid in 80% acetronitrile (ACN), and the digested peptides were dried by a SpeedVac concentrator (Savant; Holbrook, NY). The peptides were finally resuspended in 0.1% formic acid prior to MS/MS analysis.

### Identification of proteins by nanoLC-ESI-LTQ-Orbitrap MS/MS

Separation of the digested peptides was performed using EASY-nLC II (Thermo Scientific; Waltham, MA). Briefly, peptides were loaded from a cooled (7 °C) autosampler into an in-house, 3-cm-long pre-column containing 5-µm C18 resin (Dr.Maisch GmbH; Ammerbuch, Germany) and then to an in-house, 10-cm-long analytical column packed with 3-µm C18 resin (Dr.Maisch GmbH) using mobile phase A (0.1% formic acid). The peptides were then separated by mobile phase B (ACN/0.1% formic acid) gradient elution with four steps as follows: 2–9% for 15 min, 9–35% for 85 min, 35–95% for 20 min, and then 95% for 10 min at a flow rate of 200 nl/min. Peptide sequences were then analyzed by LTQ-Orbitrap-XL (Thermo Scientific) in positive mode with ESI nanosprayer ion source.

Data were acquired in a collision-induced dissociation (CID) top-12 mode under the control of the Xcalibur 2.1.0 and LTQ Tune Plus 2.5.5 software (Thermo Scientific). The cycle of one full scan was performed at a resolution of 30,000 (300–2000 *m/z*) in the Orbitrap followed by 12 data-dependent MS/MS scans in the linear ion trap with enabled preview mode for FTMS master scan. The minimum signal threshold at 1 × 10^5^ was required for a precursor ion to be selected for further fragmentation. Accumulation target values of full MS and MS/MS scan were 5 × 10^5^ and 3 × 10^4^ ions, respectively. Singly charged ions and unassigned charge states were excluded for fragmentation. Helium was used as a collision gas and the normalized collision energy was set at 35%. The activation time was 30 ms for acquiring mass spectra. The duration of dynamic exclusion was 180 s. Each sample was run in technical triplicates unless stated otherwise.

The MS/MS raw spectra were deconvoluted and then extracted into output searchable*.mgf* files using Proteome Discoverer v.1.4.1.14 software (Thermo Scientific). Mascot software version 2.4.0 (Matrix Science; London, UK) was used to search MS/MS spectra against SwissProt database of mammalian with the following standard Mascot parameters for CID: Enzyme = trypsin, maximal number of missed cleavages = 1, peptide tolerance = ± 2 ppm, MS/MS tolerance = ± 0.2 Da, fixed modification = carbamidomethyl (C), variable modification = oxidation (M), charge states = 2+ and 3+ , and decoy database on FDR < 1%.

### Bioinformatic analyses

Prediction of classical and non-classical secretory pathways was performed using SignalP 4.0 (http://www.cbs.dtu.dk/services/SignalP) and SecretomeP 2.0 (http://www.cbs.dtu.dk/services/SecretomeP) tools, respectively. Subcellular protein localization based on Gene Ontology (GO) was performed using Protein ANalysis THrough Evolutionary Relationships (PANTHER) software (http://pantherdb.org). Enrichment analysis based on GO biological process and molecular function was performed using Functional Enrichment (FunRich) analysis tool (http://www.funrich.org).

### Statistical analysis

All quantitative data are presented as mean ± SEM unless stated otherwise. Comparisons between two groups were performed using Unpaired Student’s *t*-test, whereas comparisons of different time-points within the same group were done by Paired Student’s *t-*test. Comparisons among multiple groups were performed using One-way ANOVA followed by Tukey’s post-hoc test. *p* values less than 0.05 were considered statistically significant.

## Electronic supplementary material

Supplementary Figures S1-S2

Supplementary Table S1
