# Supplementary material for: More complete polarization of renal tubular epithelial cells by artificial urine
Source: Cell Death Discov. 2018 Oct 10;4:47. doi: 10.1038/s41420-018-0112-z (PMC6180081; doi:10.1038/s41420-018-0112-z)

SUPPORTING INFORMATION

More complete polarization of renal tubular epithelial cells by artificial urine

Arada Vinaiphat, Komgrid Charngkaew and Visith Thongboonkerd*

**E-mail: thongboonkerd@dr.com (or) vthongbo@yahoo.com*

Supplementary Figure S1: Full-size scans of Western blot membranes shown as the cropped images in Figure 2. A) ZO-1; B) occludin; C) E-cadherin; D) β-catenin. GAPDH served as the loading control.

Supplementary Figure S2: Transepithelial electrical resistance (TER) of controlled and AU-assisted polarized cells in secretome study. Approximately 6.5×10^5 MDCK cells were seeded in each of a large-size (100-mm) Transwell (0.4-μm pore size) (Corning Costar; Cambridge, MA). After switching culture medium in the upper chamber for 24 h, the cells were washed with PBS^+ three times and the serum-containing medium in upper and lower chambers of the controlled group and in the lower chamber of the AU-treated group was replaced with serum-free MEM (to eliminate contaminants from serum proteins), whereas the upper chamber of the AU-treated group was refreshed with AU. The cells were further incubated at 37°C for 12 h. Finally, the resulting supernatants from the upper chambers of both groups were collected, transferred to 15-ml conical tubes, and centrifuged at 10,000 ×*g* and 4°C for 10 min. The clear supernatants were then subjected to secretome analysis. TER was periodically measured as detailed in "**Materials and Methods**". Each data point represents mean ± SEM of the data obtained from three independent biological replicates. *** $p < 0.001$ vs. control at each corresponding time-point; ### $p < 0.001$ vs. AU at 60-h.

Supplementary Figure S1

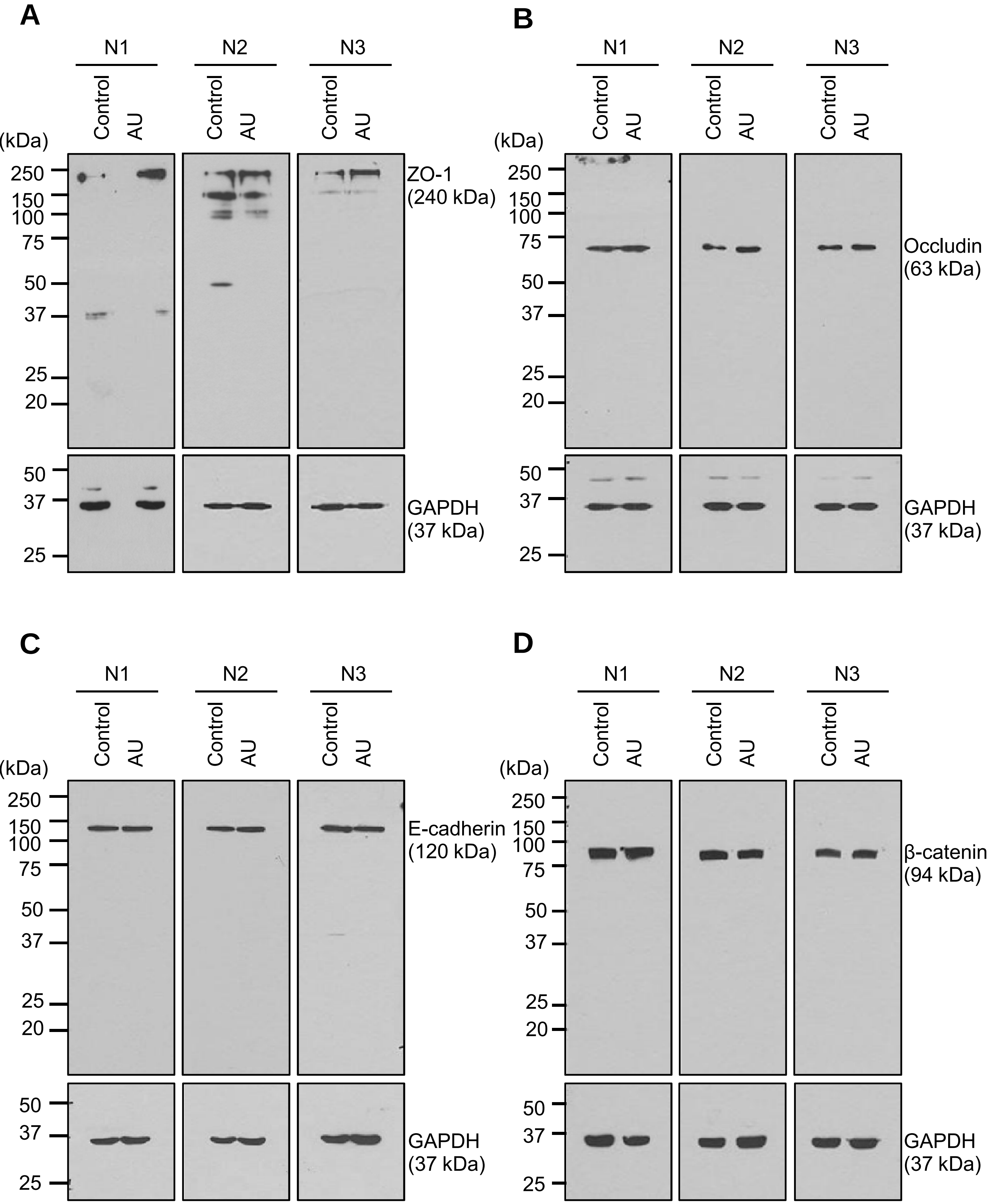

Supplementary Figure S2

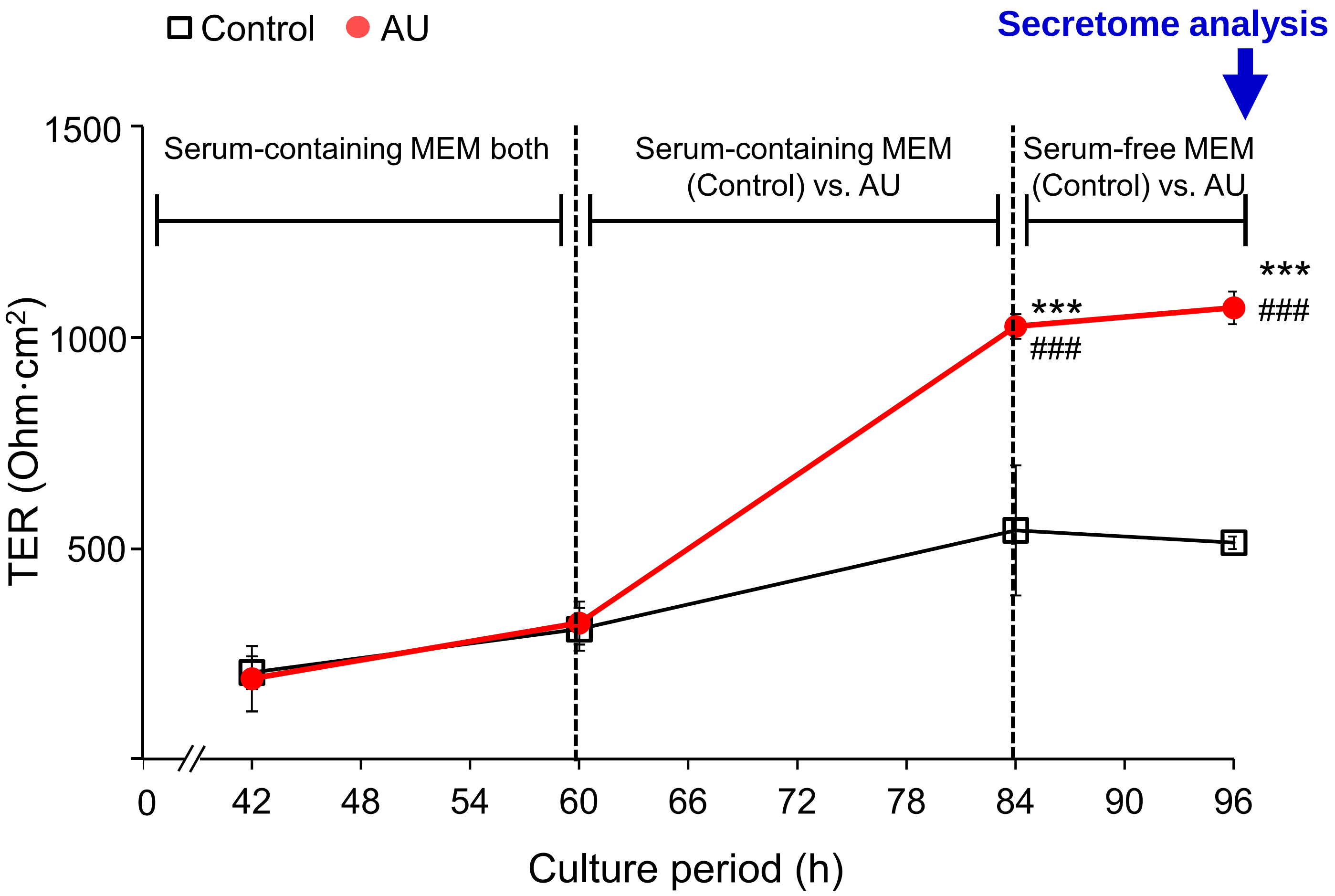

Supplement: Supplementary file 1 — Supplementary Figures S1-S2 [file 41420_2018_112_MOESM1_ESM.pdf]
