# Supplementary material for: More complete polarization of renal tubular epithelial cells by artificial urine
Source: Cell Death Discov. 2018 Oct 10;4:47. doi: 10.1038/s41420-018-0112-z (PMC6180081; doi:10.1038/s41420-018-0112-z)
Supplement: Supplementary file 2 — Supplementary Table S1 [file 41420_2018_112_MOESM2_ESM.pdf]

SUPPORTING INFORMATION

More complete polarization of renal tubular epithelial cells by artificial urine

Arada Vinaiphat, Komgrid Charngkaew, and Visith Thongboonkerd*
**E-mail: thongboonkerd@dr.com (or) vthongbo@yahoo.com*

Supplementary Table S1: Summary of proteins secreted from polarized renal tubular epithelial cells under the conventional protocol (control) vs. AU-assisted protocol by nanoLC-ESI-LTQ-Orbitrap MS/MS.

Accession number	Protein name	Identfication score	%Cov	No. of distinct/ total matched peptides	p*I*	MW (kDa)
Secreted proteins identified under the conventional (control) protocol						
P62261	14-3-3 protein epsilon	432	20.78	4/35	4.74	29.16
P61981	14-3-3 protein gamma	235	25.10	4/20	4.89	28.28
O70456	14-3-3 protein sigma	54	7.66	2/10	4.78	27.69
P29361	14-3-3 protein zeta/delta	220	24.08	4/27	4.84	27.84
P05387	60S acidic ribosomal protein P2	167	28.70	2/6	4.54	11.66
P06761	78 kDa glucose-regulated protein	141	9.94	5/9	5.16	72.30
P84856	Actin, cytoplasmic 1	3044	42.11	9/332	5.83	40.42
Q5E9B5	Actin, gamma-enteric smooth muscle	156	14.63	4/38	5.48	41.85
A7MB62	Actin-related protein 2	65	7.87	3/7	6.74	44.73
P14550	Alcohol dehydrogenase [NADP(+)]	96	6.77	1/9	6.79	36.55
P34955	Alpha-1-antiproteinase	236	3.61	1/23	6.52	46.07
P12763	Alpha-2-HS-glycoprotein	138	13.37	3/52	5.50	38.39
A5D7D1	Alpha-actinin-4	141	3.07	2/7	5.44	104.86
Q9XSJ4	Alpha-enolase	1789	35.02	10/235	6.80	47.30
Q3SZ57	Alpha-fetoprotein	62	3.77	1/3	6.29	68.54
Q06481	Amyloid-like protein 2	41	2.10	1/5	4.79	86.90
P04083	Annexin A1	64	6.07	2/4	7.02	38.69
C0HJG9	Annexin A2 (Fragments)	89	15.08	2/4	4.93	22.43
P32262	Antithrombin-III	107	3.44	1/5	6.86	52.47
Q5R7L4	Basic leucine zipper and W2 domain-containing protein 1	38	6.21	1/5	6.34	47.87
P54687	Branched-chain-amino-acid aminotransferase, cytosolic	180	3.89	1/7	5.30	42.94
P11586	C-1-tetrahydrofolate synthase, cytoplasmic	60	2.99	2/6	7.30	101.50
F1PAA9	Cadherin-1	364	3.62	2/15	4.81	97.69
P62157	Calmodulin	612	36.91	3/22	4.22	16.83
P13135	Calpain small subunit 1	39	7.22	1/2	5.20	27.91
O89001	Carboxypeptidase D	62	1.16	1/3	6.18	152.31
Q4LAL9	Cathepsin D	165	18.54	5/15	7.83	44.29
O00299	Chloride intracellular channel protein 1	311	23.65	3/16	5.17	26.91
P05371	Clusterin	76	2.68	2/9	5.71	51.34
O35142	Coatomer subunit beta'	86	2.65	2/9	5.27	102.49
P23528	Cofilin-1	382	33.13	4/24	8.09	18.49
P05997	Collagen alpha-2(V) chain	48	1.27	1/2	6.46	144.82
Q60HG7	Cystathionine gamma-lyase	104	2.72	1/10	6.96	44.49
A2Q0Z0	Elongation factor 1-alpha 1	93	11.90	3/12	9.01	50.09
P31976	Ezrin	94	7.23	3/8	6.42	68.72
Q53VB8	Ferritin light chain	182	16.00	2/9	6.00	20.08

P02751	Fibronectin	179	3.02	5/17	5.71	262.46
P21333	Filamin-A	488	6.23	10/32	6.06	280.56
O75369	Filamin-B	145	1.19	2/7	5.73	277.99
P00883	Fructose-bisphosphate aldolase A	1803	36.54	9/186	8.09	39.32
P09972	Fructose-bisphosphate aldolase C	1183	18.41	6/61	6.87	39.43
P38486	Galectin-3	107	20.27	6/45	8.06	30.31
P06396	Gelsolin	89	2.81	1/4	6.28	85.64
P08059	Glucose-6-phosphate isomerase	173	11.65	4/29	7.99	63.09
Q4KYY3	Glyceraldehyde-3-phosphate dehydrogenase	711	21.02	5/56	8.12	35.77
Q2TFN9	Heat shock 70 kDa protein 4	134	4.17	2/4	5.21	94.32
A2Q0Z1	Heat shock cognate 71 kDa protein	1145	20.43	10/62	5.52	70.85
P08238	Heat shock protein HSP 90-beta	80	4.01	2/4	5.03	83.21
Q5E9J1	Heterogeneous nuclear ribonucleoprotein F	254	8.94	3/12	5.49	45.66
O19049	Heterogeneous nuclear ribonucleoprotein K	75	2.59	1/5	5.54	50.93
Q2HJ60	Heterogeneous nuclear ribonucleoproteins A2/B1	98	10.56	4/9	8.65	35.98
Q3MHL3	Histone-binding protein RBBP4	140	10.35	3/16	4.89	47.63
Q16543	Hsp90 co-chaperone Cdc37	84	5.56	1/7	5.25	44.44
Q3KR73	KAT8 regulatory NSL complex subunit 3	41	1.25	1/6	9.33	92.98
Q8BHI4	Kelch repeat and BTB domain-containing protein 3	41	1.15	1/3	5.71	69.51
P14174	Macrophage migration inhibitory factor	89	13.91	2/4	7.88	12.47
P40121	Macrophage-capping protein	56	13.79	3/11	6.19	38.47
P11708	Malate dehydrogenase, cytoplasmic	43	6.59	2/7	6.58	36.43
P81546	Metalloproteinase inhibitor 1	230	18.84	3/14	8.44	22.84
Q50KA9	Nucleoside diphosphate kinase A	123	38.16	5/24	6.01	17.17
P62935	Peptidyl-prolyl cis-trans isomerase A	364	45.73	8/37	8.16	17.86
Q06830	Peroxiredoxin-1	399	29.65	5/33	8.13	22.10
Q9BGI1	Peroxiredoxin-5, mitochondrial	91	10.50	1/6	8.07	23.24
O35244	Peroxiredoxin-6	121	7.59	1/6	5.94	24.80
Q32PF3	PEST proteolytic signal-containing nuclear protein	39	6.18	1/16	7.49	18.98
Q3YIX4	Phosphatidylethanolamine-binding protein 1	199	22.46	3/9	7.49	20.91
P00559	Phosphoglycerate kinase 1	223	17.27	6/19	8.41	44.57
Q3SZ62	Phosphoglycerate mutase 1	258	46.85	7/34	7.18	28.83
Q15149	Plectin	168	1.37	6/33	5.96	531.47
Q5RAY0	Prefoldin subunit 5	258	11.69	1/13	6.80	17.35
Q3ZD69	Prelamin-A/C	196	5.27	4/22	7.18	74.17
P02584	Profilin-1	545	10.00	1/48	8.28	15.05
P61289	Proteasome activator complex subunit 3	55	5.91	1/3	5.95	29.49
Q3T0Y5	Proteasome subunit alpha type-2	131	14.10	2/16	7.43	25.88
P21670	Proteasome subunit alpha type-4	472	13.03	2/9	7.72	29.48
P34064	Proteasome subunit alpha type-5	91	17.43	3/8	4.86	26.37
Q2YDE4	Proteasome subunit alpha type-6	231	21.14	4/9	6.76	27.38
O14818	Proteasome subunit alpha type-7	75	10.08	2/7	8.46	27.87
P33672	Proteasome subunit beta type-3	80	16.10	2/6	6.55	22.98
Q60692	Proteasome subunit beta type-6	77	4.20	1/6	5.11	25.36
Q63009	Protein arginine N-methyltransferase 1	76	8.22	2/6	5.60	40.50
P45845	Protein-lysine 6-oxidase	52	8.03	1/4	6.44	29.05
P52480	Pyruvate kinase PKM	905	22.41	7/69	7.47	57.81
O97555	Rab GDP dissociation inhibitor alpha	77	6.26	2/4	5.14	50.49

O97556	Rab GDP dissociation inhibitor beta	152	7.64	2/9	6.47	50.29
P46940	Ras GTPase-activating-like protein IQGAP1	464	2.29	3/17	6.48	189.13
Q06AU7	Ras-related protein Rab-1B	61	7.96	1/2	5.73	22.10
P63330	Serine/threonine-protein phosphatase 2A catalytic subunit alpha isoform	64	4.21	1/4	5.54	35.59
P36873	Serine/threonine-protein phosphatase PP1-gamma catalytic subunit	277	5.57	1/18	6.54	36.96
Q29443	Serotransferrin	115	3.98	3/9	7.08	77.70
P02769	Serum albumin	409	31.63	18/90	6.18	69.25
P30626	Sorcin	29	11.11	1/7	5.59	21.66
P16086	Spectrin alpha chain, non-erythrocytic 1	319	2.14	3/14	5.33	284.46
Q9QWN8	Spectrin beta chain, non-erythrocytic 2	41	0.46	1/27	5.83	270.90
P52823	Stanniocalcin-1	690	23.89	5/36	7.99	27.60
Q3ZBZ8	Stress-induced-phosphoprotein 1	132	6.81	3/10	6.43	62.44
P10960	Sulfated glycoprotein 1	236	2.71	1/17	5.25	61.08
P78371	T-complex protein 1 subunit beta	805	20.93	6/47	6.46	57.45
P48643	T-complex protein 1 subunit epsilon	144	5.18	2/7	5.66	59.63
O77622	T-complex protein 1 subunit zeta	125	6.97	3/14	6.90	57.99
P82460	Thioredoxin	108	48.57	5/36	5.03	11.82
P07996	Thrombospondin-1	156	3.76	3/9	4.94	129.30
P37802	Transgelin-2	595	34.67	14763	8.25	22.38
P03974	Transitional endoplasmic reticulum ATPase	340	4.22	2/22	5.26	89.23
P29401	Transketolase	32	5.14	3/10	7.66	67.83
P13693	Translationally-controlled tumor protein	162	42.44	3/9	4.93	19.58
P54714	Triosephosphate isomerase	717	52.21	8/59	7.33	26.70
P02550	Tubulin alpha-1A chain	318	17.96	5/29	5.03	50.04
P81948	Tubulin alpha-4A chain	78	12.50	3/10	5.06	49.89
P02554	Tubulin beta chain	99	13.03	4/17	4.89	49.83
Q9Y333	U6 snRNA-associated Sm-like protein LSm2	102	20.00	1/2	6.52	10.83
Q0P5K3	Ubiquitin-conjugating enzyme E2 N	50	16.45	2/6	6.57	17.13
P50552	Vasodilator-stimulated phosphoprotein	51	3.68	1/7	8.94	39.81
Q3MHN5	Vitamin D-binding protein	85	6.96	2/4	5.52	53.31
Secreted proteins identified under the AU-assisted protocol						
P62261	14-3-3 protein epsilon	53	2.75	1/15	4.74	29.16
P61981	14-3-3 protein gamma	53	8.91	3/18	4.89	28.28
O70456	14-3-3 protein sigma	53	5.24	2/16	4.78	27.69
P29361	14-3-3 protein zeta/delta	53	5.31	2/17	4.84	27.84
P84856	Actin, cytoplasmic 1	343	20.78	7/53	5.83	40.42
Q5E9B5	Actin, gamma-enteric smooth muscle	343	16.49	6/47	5.48	41.85
A7MB62	Actin-related protein 2	40	1.52	1/4	6.74	44.73
P12763	Alpha-2-HS-glycoprotein	32	8.64	4/29	5.50	38.39
Q9XSJ4	Alpha-enolase	34	5.07	1/6	6.80	47.30
P50895	Basal cell adhesion molecule	41	1.43	1/3	5.81	67.36
F1PAA9	Cadherin-1	52	3.28	3/12	4.81	97.69
Q3T0Q6	Cellular nucleic acid-binding protein	137	8.24	1/10	7.71	18.73
P05371	Clusterin	33	3.8	3/11	5.71	51.34
P23528	Cofilin-1	131	19.88	4/14	8.09	18.49
P45591	Cofilin-2	131	14.46	3/11	7.88	18.70

Q28895	Epididymal secretory protein E1	284	21.48	3/42	8.02	16.05
Q1LZB9	Follistatin-related protein 3	39	4.6	1/6	7.34	27.64
P38486	Galectin-3	41	10.47	4/35	8.06	30.31
P28799	Granulins	230	3.37	2/30	6.83	63.50
O60812	Heterogeneous nuclear ribonucleoprotein C-like 1	41	3.07	1/3	5.06	32.12
Q16270	Insulin-like growth factor-binding protein 7	167	14.18	3/21	7.90	29.11
P01045	Kininogen-2	34	2.26	2/8	6.57	68.67
Q5R5W0	LIM and SH3 domain protein 1	58	3.45	1/4	7.05	29.73
P14174	Macrophage migration inhibitory factor	135	6.09	1/18	7.88	12.47
P81546	Metalloproteinase inhibitor 1	68	8.7	2/17	8.44	22.84
O15240	Neurosecretory protein VGF	157	7.64	6/44	4.78	67.22
Q32KP9	Nuclear transport factor 2	36	5.51	1/6	5.38	14.47
P13084	Nucleophosmin	35	5.48	2/6	4.77	32.54
P62935	Peptidyl-prolyl cis-trans isomerase A	54	14.02	3/13	8.16	17.86
P18203	Peptidyl-prolyl cis-trans isomerase FKBP1A	89	16.67	2/14	8.15	11.90
Q06830	Peroxiredoxin-1	89	19.6	4/13	8.13	22.10
P52552	Peroxiredoxin-2 (Fragment)	41	8.66	1/2	4.82	14.15
P06868	Plasminogen	154	7.51	6/41	7.50	91.16
P02777	Platelet factor 4	43	9.09	1/26	6.52	9.52
Q5E9A3	Poly(rC)-binding protein 1	36	7.3	2/11	7.09	37.47
P02584	Profilin-1	84	13.57	2/11	8.28	15.05
P07602	Prosaposin	44	5.53	3/7	5.17	58.07
Q60692	Proteasome subunit beta type-6	56	7.98	2/10	5.11	25.36
Q3SYX0	Protein NDRG1	203	7.55	2/13	5.49	41.60
P50543	Protein S100-A11	118	10.2	2/10	5.45	11.08
Q3ZCL8	SH3 domain-binding glutamic acid-rich-like protein 3	43	26.88	3/18	4.93	10.43
O43765	Small glutamine-rich tetratricopeptide repeat-containing protein alpha	43	4.15	1/11	4.87	34.04
P52823	Stanniocalcin-1	109	8.91	3/25	7.99	27.60
P10960	Sulfated glycoprotein 1	44	4.69	2/6	5.25	61.08
P82460	Thioredoxin	72	41.9	6/38	5.03	11.82
P37802	Transgelin-2	158	30.15	6/28	8.25	22.38
P54714	Triosephosphate isomerase	186	7.63	2/22	7.33	26.70
Q865C5	Ubiquitin	118	57.89	4/18	7.25	8.56

%Cov = %Sequence coverage [(number of the matched residues/total number of residues in the entire sequence) x 100%]
